# *N*,*N*′-Dicyclo­hexyl-*N*,*N*′-dimethyl-*N*′′-(4-nitro­benzo­yl)phospho­ric triamide

**DOI:** 10.1107/S1600536810023524

**Published:** 2010-06-23

**Authors:** Fahimeh Sabbaghi, Mahnaz Rostami Chaijan, Mehrdad Pourayoubi

**Affiliations:** a Department of Chemistry, Islamic Azad University-Zanjan Branch, PO Box 49195-467, Zanjan, Iran; b Department of Chemistry, Ferdowsi University of Mashhad, Mashhad, 91779, Iran

## Abstract

The P atom in the title compound, C_21_H_33_N_4_O_4_P, is in a slightly distorted tetra­hedral coordination environment and the phosphoryl and carbonyl groups are *anti* to each other. The environment of each N atom is essentially planar (average angles of 119.9 and 118.4°). In the crystal structure, the H atom of the C(=O)NHP(=O) group is involved in an inter­molecular –P=O⋯H–N– hydrogen bond, forming centrosymmetric dimers.

## Related literature

For applications of compounds containing the –C(=O)NHP(=O)– skeleton, see: Gholivand *et al.* (2010 ▶). For related structures, see: Pourayoubi & Sabbaghi (2009 ▶); Sabbaghi *et al.* (2010 ▶).
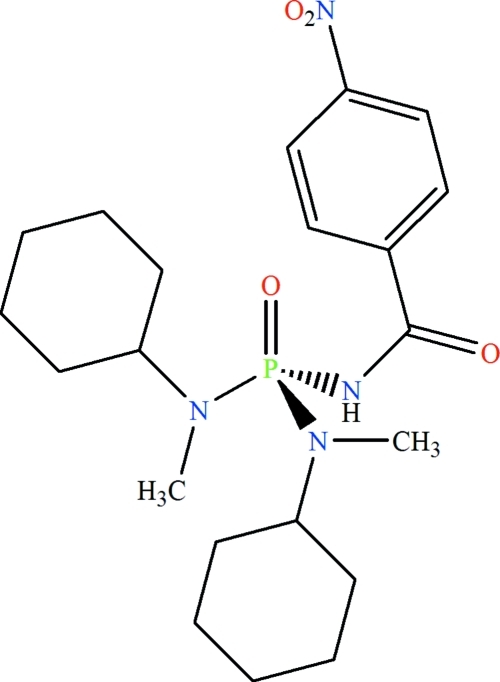


## Experimental

### 

#### Crystal data

C_21_H_33_N_4_O_4_P
*M* *_r_* = 436.48
Triclinic, 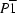

*a* = 8.6118 (16) Å
*b* = 10.838 (2) Å
*c* = 12.711 (2) Å
α = 93.089 (4)°
β = 106.792 (4)°
γ = 95.105 (3)°
*V* = 1127.4 (4) Å^3^
*Z* = 2
Mo *K*α radiation
μ = 0.16 mm^−1^
*T* = 120 K
0.40 × 0.20 × 0.20 mm

#### Data collection

Bruker SMART 1000 CCD area detector diffractometer
Absorption correction: multi-scan (*SADABS*; Sheldrick, 1998 ▶) *T*_min_ = 0.959, *T*_max_ = 0.969
12417 measured reflections
5955 independent reflections
4603 reflections with *I* > 2σ(*I*)
*R*_int_ = 0.027

#### Refinement

*R*[*F* ^2^ > 2σ(*F* ^2^)] = 0.050
*wR*(*F* ^2^) = 0.103
*S* = 1.00
5955 reflections
273 parameters
H-atom parameters constrained
Δρ_max_ = 0.39 e Å^−3^
Δρ_min_ = −0.43 e Å^−3^

### 

Data collection: *SMART* (Bruker, 1998 ▶); cell refinement: *SAINT-Plus* (Bruker, 1998 ▶); data reduction: *SAINT-Plus*; program(s) used to solve structure: *SHELXTL* (Sheldrick, 2008 ▶); program(s) used to refine structure: *SHELXTL*; molecular graphics: *SHELXTL*; software used to prepare material for publication: *SHELXTL*.

## Supplementary Material

Crystal structure: contains datablocks I, global. DOI: 10.1107/S1600536810023524/lh5061sup1.cif

Structure factors: contains datablocks I. DOI: 10.1107/S1600536810023524/lh5061Isup2.hkl

Additional supplementary materials:  crystallographic information; 3D view; checkCIF report

## Figures and Tables

**Table 1:** Hydrogen-bond geometry (Å, °)

*D*—H⋯*A*	*D*—H	H⋯*A*	*D*⋯*A*	*D*—H⋯*A*
N1—H1*N*⋯O1^i^	0.86	1.91	2.7622 (18)	167

Symmetry code: (i) 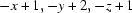.

**Fig. 1.**
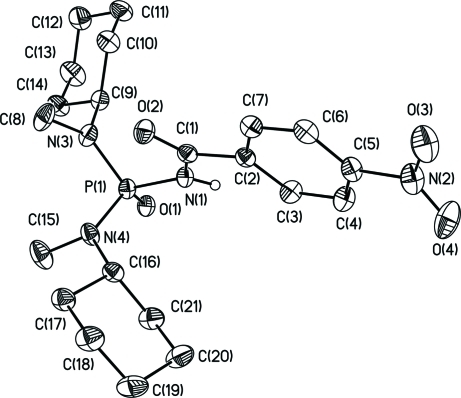
A view of the title compound, showing the atom-numbering scheme. Displacement ellipsoids are drawn at the 50% probability level (H(C) atoms are omitted for clarity).

**Fig. 2.**
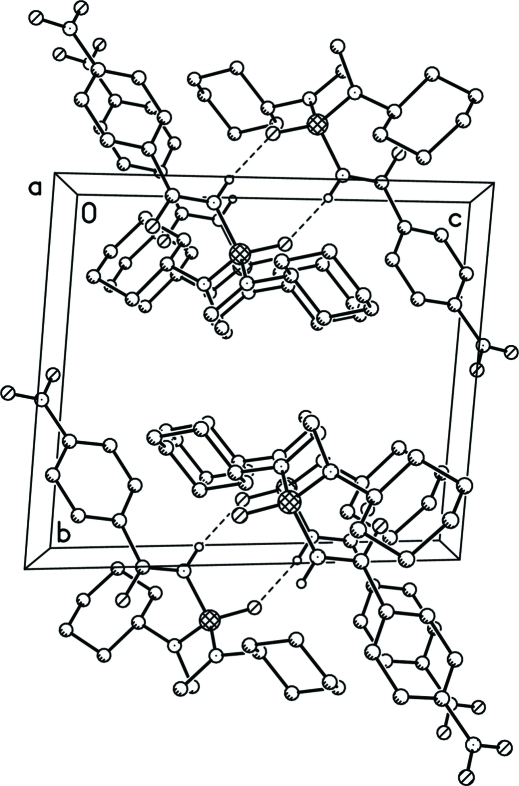
Part of the crystal structure of the title compound viewed approximately along the a axis showing centrosymmetric H-bonded (dashed lines) dimers. Only H atoms involved in hydrogen bonds are shown.

## supplementary crystallographic information

### Comment

Carbacylamidophosphates with a –C(O)NHP(O)– skeleton have attracted attention because of their roles as the *O*,*O*'-donor ligands for metal complexation (Gholivand *et al.*, 2010). In the previous work, the structures of two compounds with a P(O)[NHC(O)C_6_H_4_(4-NO_2_)] moiety have been investigated: P(O)[NHC(O)C_6_H_4_(4-NO_2_)][N(CH(CH_3_)_2_)(CH_2_C_6_H_5_)]_2_ (Pourayoubi & Sabbaghi, 2009) and P(O)[NHC(O)C_6_H_4_(4-NO_2_)][NHC_6_H_11_]_2_ (Sabbaghi *et al.*, 2010). Here, we report the synthesis and crystal structure of a third compound, P(O)[NHC(O)C_6_H_4_(4-NO_2_)][N(CH_3_)(C_6_H_11_)]_2_. The phosphoryl and carbonyl groups are *anti* to each other and the phosphorus atom has a slightly distorted tetrahedral configuration (Fig 1). The bond angles around the P atom are in the range of 104.81 (7)°-117.28 (8)°. The P1–N3 and P1–N4 bond lengths (1.6315 (15) Å and 1.6446 (15) Å) are shorter than the P1–N1 bond (1.6859 (14) Å). The environment of the nitrogen atoms is essentially planar; the angles C8–N3–P1, C8–N3–C9 and P1–N3–C9 are 124.25 (12)°, 117.46 (14)° and 118.02 (11)°, respectively (with average = 119.9°). A similar result was obtained for the bond angles around N4 atom (average = 118.4°). Furthermore, the angle C1–N1–P1 is 125.20 (12)°. The P═O bond length of 1.4834 (13) Å is standard for phosphoramidate compounds. The hydrogen atom of the C(═O)NHP(═O) group is involved in an intermolecular –P═O···H—N– hydrogen bond (see Table 1) to form a centrosymmetric dimeric aggregate. A view of crystal packing along the *a*  axis is shown in Fig. 2.

### Experimental

4-NO_2_—C_6_H_4_C(O)NHP(O)Cl_2_ was prepared according to the procedure of literature (Sabbaghi *et al.*, 2010). To a solution of (0.566 g, 2 mmol) 4-NO_2_C_6_H_4_C(O)NHP(O)Cl_2_ in CH_3_CN (20 ml), a solution of *N*-methylcyclohexylamine (0.906 g, 8 mmol) in CH_3_CN (5 ml) was added dropwise at 273K. After 4 h the solvent was removed in vacuum. Single crystals were obtained from a solution of title compound in CH_3_CN and n-C_6_H_14_ (4:1) after slow evaporation at room temperature. IR (KBr, cm^-1^): 3063, 2930, 2855, 1685, 1523, 1453, 1340, 1267, 1183, 1106, 1004, 848, 712.

### Refinement

The hydrogen atom of the NH group was seen in a difference Fourier map and included with N-H = 0.86Å. The other H atoms were placed in calculated positions C-H = 0.95-1.00Å. All hydrogen atoms were refined in a riding-model approximation with *U*_iso_(H) parameters equal to 1.2 *U*_eq_(Ci), or for methyl groups equal to 1.5 *U*_eq_(Cii), where U(Ci) and U(Cii) are respectively the equivalent thermal parameters of the carbon atoms to which corresponding H atoms are bonded.

### Crystal data

**Table:**

C_21_H_33_N_4_O_4_P	*Z* = 2
*M**_r_* = 436.48	*F*(000) = 468
Triclinic, *P*1	*D*_x_ = 1.286 Mg m^−^^3^
Hall symbol: -P 1	Mo *K*α radiation, λ = 0.71073 Å
*a* = 8.6118 (16) Å	Cell parameters from 2045 reflections
*b* = 10.838 (2) Å	θ = 2–25°
*c* = 12.711 (2) Å	µ = 0.16 mm^−^^1^
α = 93.089 (4)°	*T* = 120 K
β = 106.792 (4)°	Prism, colorless
γ = 95.105 (3)°	0.40 × 0.20 × 0.20 mm
*V* = 1127.4 (4) Å^3^	

### Data collection

**Table:**

Bruker SMART 1000 CCD area detector diffractometer	5955 independent reflections
Radiation source: fine-focus sealed tube	4603 reflections with *I* > 2σ(*I*)
graphite	*R*_int_ = 0.027
φ and ω scans	θ_max_ = 29.0°, θ_min_ = 1.7°
Absorption correction: multi-scan (*SADABS*; Sheldrick, 1998)	*h* = −11→11
*T*_min_ = 0.959, *T*_max_ = 0.969	*k* = −14→14
12417 measured reflections	*l* = −17→17

### Refinement

**Table:**

Refinement on *F*^2^	Primary atom site location: structure-invariant direct methods
Least-squares matrix: full	Secondary atom site location: difference Fourier map
*R*[*F*^2^ > 2σ(*F*^2^)] = 0.050	Hydrogen site location: mixed
*wR*(*F*^2^) = 0.103	H-atom parameters constrained
*S* = 1.00	*w* = 1/[σ^2^(*F*_o_^2^) + (0.001*P*)^2^ + 1.3*P*] where *P* = (*F*_o_^2^ + 2*F*_c_^2^)/3
5955 reflections	(Δ/σ)_max_ = 0.001
273 parameters	Δρ_max_ = 0.39 e Å^−^^3^
0 restraints	Δρ_min_ = −0.43 e Å^−^^3^

### Special details

**Table:**

Geometry. All e.s.d.'s (except the e.s.d. in the dihedral angle between two l.s. planes) are estimated using the full covariance matrix. The cell e.s.d.'s are taken into account individually in the estimation of e.s.d.'s in distances, angles and torsion angles; correlations between e.s.d.'s in cell parameters are only used when they are defined by crystal symmetry. An approximate (isotropic) treatment of cell e.s.d.'s is used for estimating e.s.d.'s involving l.s. planes.
Refinement. Refinement of *F*^2^ against ALL reflections. The weighted *R*-factor *wR* and goodness of fit *S* are based on *F*^2^, conventional *R*-factors *R* are based on *F*, with *F* set to zero for negative *F*^2^. The threshold expression of *F*^2^ > σ(*F*^2^) is used only for calculating *R*-factors(gt) *etc*. and is not relevant to the choice of reflections for refinement. *R*-factors based on *F*^2^ are statistically about twice as large as those based on *F*, and *R*- factors based on ALL data will be even larger.

### Fractional atomic coordinates and isotropic or equivalent isotropic displacement parameters (Å^2^)

**Table:**

	*x*	*y*	*z*	*U*_iso_*/*U*_eq_	
P1	0.45851 (5)	0.82031 (4)	0.58020 (3)	0.02026 (10)	
O1	0.48700 (15)	0.85007 (11)	0.47427 (9)	0.0235 (3)	
O2	0.37428 (15)	0.88786 (11)	0.78908 (10)	0.0269 (3)	
O3	0.60736 (19)	1.44437 (13)	1.10559 (11)	0.0390 (3)	
O4	0.7761 (2)	1.49349 (14)	1.01319 (12)	0.0453 (4)	
N1	0.47053 (17)	0.95776 (12)	0.65239 (11)	0.0199 (3)	
H1N	0.4965	1.0218	0.6211	0.024*	
N2	0.6661 (2)	1.42572 (14)	1.02941 (12)	0.0305 (3)	
N3	0.28286 (18)	0.73684 (13)	0.55664 (11)	0.0240 (3)	
N4	0.59191 (18)	0.74205 (13)	0.66231 (12)	0.0247 (3)	
C1	0.43680 (19)	0.97284 (15)	0.75065 (13)	0.0200 (3)	
C2	0.48973 (19)	1.09818 (15)	0.81576 (13)	0.0197 (3)	
C3	0.5913 (2)	1.19046 (16)	0.78890 (14)	0.0246 (3)	
H3A	0.6219	1.1794	0.7232	0.030*	
C4	0.6481 (2)	1.29926 (16)	0.85839 (14)	0.0263 (4)	
H4A	0.7182	1.3626	0.8413	0.032*	
C5	0.5999 (2)	1.31276 (15)	0.95283 (13)	0.0232 (3)	
C6	0.4977 (2)	1.22431 (16)	0.98073 (14)	0.0244 (3)	
H6A	0.4653	1.2369	1.0455	0.029*	
C7	0.4429 (2)	1.11573 (16)	0.91131 (13)	0.0229 (3)	
H7A	0.3729	1.0528	0.9292	0.027*	
C8	0.2412 (2)	0.65393 (18)	0.63379 (15)	0.0335 (4)	
H8A	0.1906	0.5735	0.5943	0.050*	
H8B	0.1646	0.6909	0.6668	0.050*	
H8C	0.3405	0.6418	0.6920	0.050*	
C9	0.1508 (2)	0.75515 (15)	0.45520 (13)	0.0217 (3)	
H9A	0.1909	0.8281	0.4217	0.026*	
C10	−0.0048 (2)	0.78658 (18)	0.47985 (15)	0.0306 (4)	
H10A	0.0200	0.8621	0.5318	0.037*	
H10B	−0.0464	0.7174	0.5156	0.037*	
C11	−0.1361 (2)	0.80878 (19)	0.37426 (16)	0.0342 (4)	
H11A	−0.2379	0.8235	0.3920	0.041*	
H11B	−0.0995	0.8839	0.3430	0.041*	
C12	−0.1699 (2)	0.69742 (19)	0.28905 (17)	0.0382 (5)	
H12A	−0.2174	0.6243	0.3172	0.046*	
H12B	−0.2504	0.7160	0.2202	0.046*	
C13	−0.0139 (3)	0.6671 (2)	0.26434 (16)	0.0393 (5)	
H13A	0.0280	0.7371	0.2296	0.047*	
H13B	−0.0382	0.5923	0.2116	0.047*	
C14	0.1171 (2)	0.64358 (17)	0.37005 (15)	0.0297 (4)	
H14A	0.0798	0.5685	0.4010	0.036*	
H14B	0.2189	0.6285	0.3525	0.036*	
C15	0.6164 (3)	0.62277 (18)	0.61104 (17)	0.0404 (5)	
H15A	0.6089	0.5568	0.6597	0.061*	
H15B	0.7243	0.6294	0.5993	0.061*	
H15C	0.5321	0.6029	0.5400	0.061*	
C16	0.7269 (2)	0.79996 (16)	0.75819 (13)	0.0226 (3)	
H16A	0.6797	0.8608	0.7989	0.027*	
C17	0.7955 (2)	0.70390 (17)	0.83827 (14)	0.0279 (4)	
H17A	0.8455	0.6425	0.8016	0.034*	
H17B	0.7059	0.6590	0.8601	0.034*	
C18	0.9245 (2)	0.76902 (19)	0.94149 (15)	0.0329 (4)	
H18A	0.8718	0.8248	0.9816	0.040*	
H18B	0.9714	0.7059	0.9913	0.040*	
C19	1.0606 (2)	0.8443 (2)	0.91066 (16)	0.0363 (5)	
H19A	1.1212	0.7874	0.8780	0.044*	
H19B	1.1380	0.8891	0.9780	0.044*	
C20	0.9923 (2)	0.9376 (2)	0.82845 (17)	0.0361 (5)	
H20A	1.0822	0.9815	0.8063	0.043*	
H20B	0.9424	1.0003	0.8640	0.043*	
C21	0.8637 (2)	0.87251 (19)	0.72583 (15)	0.0306 (4)	
H21A	0.8179	0.9352	0.6752	0.037*	
H21B	0.9156	0.8150	0.6866	0.037*	

### Atomic displacement parameters (Å^2^)

**Table:**

	*U*^11^	*U*^22^	*U*^33^	*U*^12^	*U*^13^	*U*^23^
P1	0.0236 (2)	0.0180 (2)	0.01684 (19)	0.00119 (16)	0.00277 (16)	0.00079 (15)
O1	0.0288 (6)	0.0206 (6)	0.0198 (6)	0.0005 (5)	0.0062 (5)	−0.0009 (4)
O2	0.0322 (7)	0.0247 (6)	0.0249 (6)	0.0000 (5)	0.0108 (5)	0.0037 (5)
O3	0.0589 (10)	0.0343 (8)	0.0216 (6)	0.0068 (7)	0.0091 (6)	−0.0052 (5)
O4	0.0593 (10)	0.0356 (8)	0.0337 (8)	−0.0152 (7)	0.0101 (7)	−0.0085 (6)
N1	0.0244 (7)	0.0173 (6)	0.0174 (6)	0.0013 (5)	0.0058 (5)	0.0014 (5)
N2	0.0421 (9)	0.0260 (8)	0.0192 (7)	0.0045 (7)	0.0025 (7)	−0.0004 (6)
N3	0.0262 (7)	0.0240 (7)	0.0177 (7)	−0.0025 (6)	0.0004 (6)	0.0062 (5)
N4	0.0282 (8)	0.0191 (7)	0.0223 (7)	0.0061 (6)	−0.0002 (6)	−0.0017 (5)
C1	0.0182 (7)	0.0223 (8)	0.0181 (7)	0.0043 (6)	0.0023 (6)	0.0028 (6)
C2	0.0193 (8)	0.0213 (8)	0.0172 (7)	0.0047 (6)	0.0028 (6)	0.0011 (6)
C3	0.0277 (9)	0.0273 (9)	0.0186 (8)	−0.0001 (7)	0.0075 (7)	0.0002 (6)
C4	0.0310 (9)	0.0256 (9)	0.0203 (8)	−0.0020 (7)	0.0058 (7)	0.0010 (7)
C5	0.0274 (9)	0.0218 (8)	0.0164 (7)	0.0046 (7)	0.0001 (6)	−0.0009 (6)
C6	0.0280 (9)	0.0282 (9)	0.0176 (8)	0.0082 (7)	0.0063 (7)	0.0025 (6)
C7	0.0243 (8)	0.0246 (8)	0.0199 (8)	0.0042 (7)	0.0061 (6)	0.0033 (6)
C8	0.0365 (10)	0.0313 (10)	0.0265 (9)	−0.0097 (8)	0.0021 (8)	0.0110 (7)
C9	0.0236 (8)	0.0219 (8)	0.0166 (7)	0.0012 (6)	0.0014 (6)	0.0028 (6)
C10	0.0325 (10)	0.0333 (10)	0.0259 (9)	0.0075 (8)	0.0077 (8)	0.0015 (7)
C11	0.0286 (10)	0.0361 (10)	0.0372 (11)	0.0117 (8)	0.0058 (8)	0.0063 (8)
C12	0.0287 (10)	0.0354 (11)	0.0394 (11)	0.0051 (8)	−0.0076 (8)	0.0013 (9)
C13	0.0389 (11)	0.0436 (12)	0.0246 (9)	0.0118 (9)	−0.0075 (8)	−0.0085 (8)
C14	0.0275 (9)	0.0305 (9)	0.0250 (9)	0.0079 (7)	−0.0021 (7)	−0.0045 (7)
C15	0.0504 (13)	0.0275 (10)	0.0350 (11)	0.0144 (9)	−0.0020 (9)	−0.0058 (8)
C16	0.0219 (8)	0.0252 (8)	0.0189 (8)	0.0050 (6)	0.0024 (6)	0.0005 (6)
C17	0.0269 (9)	0.0315 (9)	0.0246 (9)	0.0057 (7)	0.0048 (7)	0.0068 (7)
C18	0.0291 (10)	0.0429 (11)	0.0238 (9)	0.0041 (8)	0.0019 (7)	0.0091 (8)
C19	0.0229 (9)	0.0521 (13)	0.0301 (10)	0.0038 (8)	0.0011 (8)	0.0071 (9)
C20	0.0248 (9)	0.0440 (12)	0.0354 (10)	−0.0041 (8)	0.0039 (8)	0.0093 (9)
C21	0.0274 (9)	0.0392 (10)	0.0259 (9)	0.0048 (8)	0.0074 (7)	0.0100 (8)

### Geometric parameters (Å, °)

**Table:**

P1—O1	1.4834 (13)	C10—H10B	0.9900
P1—N3	1.6315 (15)	C11—C12	1.526 (3)
P1—N4	1.6446 (15)	C11—H11A	0.9900
P1—N1	1.6859 (14)	C11—H11B	0.9900
O2—C1	1.220 (2)	C12—C13	1.524 (3)
O3—N2	1.231 (2)	C12—H12A	0.9900
O4—N2	1.219 (2)	C12—H12B	0.9900
N1—C1	1.366 (2)	C13—C14	1.534 (2)
N1—H1N	0.8628	C13—H13A	0.9900
N2—C5	1.483 (2)	C13—H13B	0.9900
N3—C8	1.462 (2)	C14—H14A	0.9900
N3—C9	1.489 (2)	C14—H14B	0.9900
N4—C15	1.476 (2)	C15—H15A	0.9800
N4—C16	1.483 (2)	C15—H15B	0.9800
C1—C2	1.513 (2)	C15—H15C	0.9800
C2—C3	1.391 (2)	C16—C21	1.525 (2)
C2—C7	1.395 (2)	C16—C17	1.531 (2)
C3—C4	1.395 (2)	C16—H16A	1.0000
C3—H3A	0.9500	C17—C18	1.540 (3)
C4—C5	1.383 (2)	C17—H17A	0.9900
C4—H4A	0.9500	C17—H17B	0.9900
C5—C6	1.374 (2)	C18—C19	1.524 (3)
C6—C7	1.391 (2)	C18—H18A	0.9900
C6—H6A	0.9500	C18—H18B	0.9900
C7—H7A	0.9500	C19—C20	1.525 (3)
C8—H8A	0.9800	C19—H19A	0.9900
C8—H8B	0.9800	C19—H19B	0.9900
C8—H8C	0.9800	C20—C21	1.534 (3)
C9—C10	1.523 (2)	C20—H20A	0.9900
C9—C14	1.527 (2)	C20—H20B	0.9900
C9—H9A	1.0000	C21—H21A	0.9900
C10—C11	1.531 (3)	C21—H21B	0.9900
C10—H10A	0.9900		
			
O1—P1—N3	109.91 (7)	H11A—C11—H11B	108.0
O1—P1—N4	117.28 (8)	C13—C12—C11	111.06 (16)
N3—P1—N4	105.39 (8)	C13—C12—H12A	109.4
O1—P1—N1	106.20 (7)	C11—C12—H12A	109.4
N3—P1—N1	113.38 (8)	C13—C12—H12B	109.4
N4—P1—N1	104.81 (7)	C11—C12—H12B	109.4
C1—N1—P1	125.20 (12)	H12A—C12—H12B	108.0
C1—N1—H1N	120.1	C12—C13—C14	111.07 (17)
P1—N1—H1N	114.6	C12—C13—H13A	109.4
O4—N2—O3	124.33 (16)	C14—C13—H13A	109.4
O4—N2—C5	117.72 (15)	C12—C13—H13B	109.4
O3—N2—C5	117.95 (16)	C14—C13—H13B	109.4
C8—N3—C9	117.46 (14)	H13A—C13—H13B	108.0
C8—N3—P1	124.25 (12)	C9—C14—C13	110.50 (15)
C9—N3—P1	118.02 (11)	C9—C14—H14A	109.5
C15—N4—C16	116.81 (14)	C13—C14—H14A	109.5
C15—N4—P1	114.39 (12)	C9—C14—H14B	109.5
C16—N4—P1	124.01 (11)	C13—C14—H14B	109.5
O2—C1—N1	122.38 (15)	H14A—C14—H14B	108.1
O2—C1—C2	119.89 (15)	N4—C15—H15A	109.5
N1—C1—C2	117.61 (14)	N4—C15—H15B	109.5
C3—C2—C7	119.80 (15)	H15A—C15—H15B	109.5
C3—C2—C1	122.95 (15)	N4—C15—H15C	109.5
C7—C2—C1	117.06 (15)	H15A—C15—H15C	109.5
C2—C3—C4	119.99 (16)	H15B—C15—H15C	109.5
C2—C3—H3A	120.0	N4—C16—C21	113.46 (14)
C4—C3—H3A	120.0	N4—C16—C17	111.50 (14)
C5—C4—C3	118.47 (16)	C21—C16—C17	110.48 (14)
C5—C4—H4A	120.8	N4—C16—H16A	107.0
C3—C4—H4A	120.8	C21—C16—H16A	107.0
C6—C5—C4	122.96 (16)	C17—C16—H16A	107.0
C6—C5—N2	118.42 (15)	C16—C17—C18	110.00 (15)
C4—C5—N2	118.59 (16)	C16—C17—H17A	109.7
C5—C6—C7	118.05 (16)	C18—C17—H17A	109.7
C5—C6—H6A	121.0	C16—C17—H17B	109.7
C7—C6—H6A	121.0	C18—C17—H17B	109.7
C6—C7—C2	120.72 (16)	H17A—C17—H17B	108.2
C6—C7—H7A	119.6	C19—C18—C17	111.18 (16)
C2—C7—H7A	119.6	C19—C18—H18A	109.4
N3—C8—H8A	109.5	C17—C18—H18A	109.4
N3—C8—H8B	109.5	C19—C18—H18B	109.4
H8A—C8—H8B	109.5	C17—C18—H18B	109.4
N3—C8—H8C	109.5	H18A—C18—H18B	108.0
H8A—C8—H8C	109.5	C18—C19—C20	111.10 (16)
H8B—C8—H8C	109.5	C18—C19—H19A	109.4
N3—C9—C10	112.56 (14)	C20—C19—H19A	109.4
N3—C9—C14	111.46 (14)	C18—C19—H19B	109.4
C10—C9—C14	111.44 (15)	C20—C19—H19B	109.4
N3—C9—H9A	107.0	H19A—C19—H19B	108.0
C10—C9—H9A	107.0	C19—C20—C21	111.01 (17)
C14—C9—H9A	107.0	C19—C20—H20A	109.4
C9—C10—C11	111.17 (15)	C21—C20—H20A	109.4
C9—C10—H10A	109.4	C19—C20—H20B	109.4
C11—C10—H10A	109.4	C21—C20—H20B	109.4
C9—C10—H10B	109.4	H20A—C20—H20B	108.0
C11—C10—H10B	109.4	C16—C21—C20	110.50 (15)
H10A—C10—H10B	108.0	C16—C21—H21A	109.5
C12—C11—C10	111.01 (16)	C20—C21—H21A	109.5
C12—C11—H11A	109.4	C16—C21—H21B	109.5
C10—C11—H11A	109.4	C20—C21—H21B	109.5
C12—C11—H11B	109.4	H21A—C21—H21B	108.1
C10—C11—H11B	109.4		
			
O1—P1—N1—C1	173.62 (13)	C4—C5—C6—C7	1.1 (3)
N3—P1—N1—C1	52.83 (15)	N2—C5—C6—C7	−176.72 (15)
N4—P1—N1—C1	−61.59 (15)	C5—C6—C7—C2	−0.6 (2)
O1—P1—N3—C8	156.29 (15)	C3—C2—C7—C6	−0.5 (2)
N4—P1—N3—C8	29.03 (17)	C1—C2—C7—C6	174.68 (15)
N1—P1—N3—C8	−85.04 (17)	C8—N3—C9—C10	49.4 (2)
O1—P1—N3—C9	−29.95 (15)	P1—N3—C9—C10	−124.79 (14)
N4—P1—N3—C9	−157.20 (12)	C8—N3—C9—C14	−76.7 (2)
N1—P1—N3—C9	88.73 (13)	P1—N3—C9—C14	109.15 (15)
O1—P1—N4—C15	−55.86 (16)	N3—C9—C10—C11	178.22 (15)
N3—P1—N4—C15	66.78 (16)	C14—C9—C10—C11	−55.7 (2)
N1—P1—N4—C15	−173.32 (14)	C9—C10—C11—C12	55.4 (2)
O1—P1—N4—C16	98.72 (15)	C10—C11—C12—C13	−55.9 (2)
N3—P1—N4—C16	−138.64 (14)	C11—C12—C13—C14	56.5 (2)
N1—P1—N4—C16	−18.74 (16)	N3—C9—C14—C13	−177.44 (16)
P1—N1—C1—O2	−9.5 (2)	C10—C9—C14—C13	55.9 (2)
P1—N1—C1—C2	166.63 (11)	C12—C13—C14—C9	−56.2 (2)
O2—C1—C2—C3	166.11 (16)	C15—N4—C16—C21	81.6 (2)
N1—C1—C2—C3	−10.1 (2)	P1—N4—C16—C21	−72.46 (19)
O2—C1—C2—C7	−8.9 (2)	C15—N4—C16—C17	−43.9 (2)
N1—C1—C2—C7	174.87 (14)	P1—N4—C16—C17	162.03 (13)
C7—C2—C3—C4	1.1 (3)	N4—C16—C17—C18	−174.96 (15)
C1—C2—C3—C4	−173.82 (16)	C21—C16—C17—C18	57.9 (2)
C2—C3—C4—C5	−0.5 (3)	C16—C17—C18—C19	−56.7 (2)
C3—C4—C5—C6	−0.6 (3)	C17—C18—C19—C20	55.6 (2)
C3—C4—C5—N2	177.27 (16)	C18—C19—C20—C21	−55.6 (2)
O4—N2—C5—C6	168.44 (17)	N4—C16—C21—C20	175.75 (15)
O3—N2—C5—C6	−11.0 (2)	C17—C16—C21—C20	−58.2 (2)
O4—N2—C5—C4	−9.5 (2)	C19—C20—C21—C16	56.9 (2)
O3—N2—C5—C4	171.09 (16)		

### Hydrogen-bond geometry (Å, °)

**Table:**

*D*—H···*A*	*D*—H	H···*A*	*D*···*A*	*D*—H···*A*
N1—H1N···O1^i^	0.86	1.91	2.7622 (18)	167

Symmetry codes: (i) −*x*+1, −*y*+2, −*z*+1.
